# What Do We Need to Do for Better Casualty Support in Disasters?

**Published:** 2011-07-01

**Authors:** M Nekooei Moghadam, S Saeed, N Khanjani, M Arab

**Affiliations:** 1 Kerman University of Medical Sciences, Kerman, Iran

**Keywords:** Disaster relief planning, Mass casualty incidents, Nurses, Relief work

Dear Editor,

Statistics show that Iran is one of the main disaster prone countries[1]. In the Bam earthquake alone in December 26, 2003, more than 25000 people died, 50,000 were injured and 100,000 lost their homes[2]. Disaster may happen during any person’s life and preparedness is the best way for preventing the dra-matic consequences. By sure, implementing proper preparedness for disasters in developing countries with high populations and low resources is difficult. One task for disaster preparedness is to find out the right people to engage in relief operations and to determine how and what tasks they should perform.

Despite the allocation of tremendous resources and finances for educating and simulating disaster response situations in many countries, the actual dis-aster response, even in developed countries has been inadequate and recently there has been renewed emphasis on better planning and research to improve medical responses to sudden and catastrophic events.[3]

In Iran, there are many experienced nurses and aid workers working in educational hospitals, with valuable information and experience about aid and casualty support after disasters. Our team interviewed and documented the valuable experiences of more than 20 nurses in Kerman through a qualitative descriptive phenomenology study. The results helped us in understanding the shortcomings of aid and casualty support, and suggesting the proper interventions, educational and technical programs for more effective dis-aster management in the future.

The most frequent topic mentioned in our data were the facts that some caregivers could not cope with the mental and psychological burden and stress of post disaster care. They commented that aid workers should be selected among those who have strong personalities, dedication, energy and physical strength. Other studies have also commented that not all persons can or should be recruited as first line caregivers[4]. The aid workers should be selected in advance and should be trained intensively and frequently in both theoretical and practical aspects of casualty aid through specific well organized educational programs to stay ready and up to date. Our participating nurses, although were experienced but they felt to need the knowledge and practice more about trauma complications and trauma care; and needed frequent workshops, seminars, well organized and well conducted drills and etc. Some commented that untrained volunteers should not enter the circle of aid workers; because they caused chaos and various problems. They suggested clear planning and their duties and scope of responsibility were determined in advance clearly and also each individual’s training should be planned properly according to its duties and an organized schedule to perform accordingly in dis-asters. Participants also suggested the trained aid workers should be registered, accessible and should enter the scene immediately without time waste and going through bureaucracy.

All of the nurses referred to the crucial role of strong and well trained managers in disaster control, as weak management after the Bam disaster led to some chaos and inefficient use of human, financial and physical resources. The participants believed that the proper amount of equipment and facilities should be prepared, stored and checked in advance and prompt access should be possible. The nurses’ comments also raised the important issue of allocating predetermined places with specified admission capacities such as halls and stadiums for casualty accommodation and practicing transfer to these places and patient care onsite during the drills.

Our participants suggested that even now and in ordinary circumstances there are not enough nurses and caregivers in the hospital wards, and if a disaster happens, the situation is going to be even worse. They were worried that if another disaster such as the Bam earthquake happens, they would face serious problems and shortcomings. Eventually, many nurses con-fessed that they are not mentally, educationally and physically prepared for the next disaster and the per-formed drills are inadequate and should be revised. They also recommended extensively on how proper drills should be performed and the necessary equipment that should be available and caregivers need to know how to work with.

Other comments included emphasis on aid workers taking care of personal safety and the importance of region security, speed and accuracy, the crucial role of central leadership, order, management, communication skills, documenting, exchanging and re-porting patient and general information, learning legal and ethical considerations and etc. Similar to other studies, our participants also suggested the necessity for proper planning to provide the basic facilities for their safe accommodation, rest, water and food supply and their families’ support[5][6].

Other studies have also highlighted the problem of scarce resources, inefficient management for casualty care, high mental and psychological stresses that caregivers go through and the necessity of education and preparedness[6][7][8][9][10]. We also noticed that some obstacles to aid workers preparedness in our study were their heavy work load, time constraints, financial limitations, and competing demands. Confirming some of the results of previous studies, this study highlights many pitfalls and areas of concern for post disaster casualty support in Iran.
